# A Novel Use of an Electronic Differential Diagnosis Generator in the Emergency Department Setting

**DOI:** 10.7759/cureus.34211

**Published:** 2023-01-25

**Authors:** Erica L Burkett, Brett R Todd

**Affiliations:** 1 Department of Emergency Medicine, Beaumont Hospital, Royal Oak, USA

**Keywords:** emergency medicine training, diagnostic error, documentation workflow, healthcare technology, differential diagnosis

## Abstract

Introduction

The ability of electronic differential diagnosis (DDx) tools to generate accurate diagnoses has been well established in simulation and primary care clinical environments. However, the use of such tools has not been well studied in the emergency department (ED). We aimed to characterize the use and perceptions of a DDx tool among emergency medicine (EM) clinicians who were newly provided with access to such a tool.

Methods

We performed a pilot study investigating the utilization of a DDx tool by clinicians in an ED setting shortly after the tool was introduced. After six months of use, retrospective data were analyzed to characterize the use of the tool among ED clinicians. The clinicians were also surveyed on their perceptions of the use of the tool in the ED setting.

Results

There were 224 total queries, which were regarding 107 unique patients. The most searched symptoms were related to constitutional, dermatologic, and gastrointestinal complaints whereas symptoms related to toxicology and trauma were less commonly searched. Survey respondents rated the tool favorably, and when not used, reported reasons including forgetting that the tool was available for use, not feeling the need to use the tool, and disruption to workflow.

Conclusions

Electronic DDx tools may have some utility in assisting ED clinicians in generating a DDx, however, clinician adoption and workflow integration are barriers to their utility.

## Introduction

This article was previously presented as a poster at the 2022 American Academy of Emergency Medicine (AAEM) Annual Scientific Assembly on April 26, 2022.

Background

The ability of differential diagnosis (DDx) tools to generate an accurate DDx compared to gold standard diagnoses has been demonstrated in several retrospective chart reviews and case series from primary care settings [1,2]. However, studies demonstrating the accuracy and efficacy of DDx tools in real-time clinical situations are sparse with high heterogeneity in their methods and occurrences of conflict of interest via financial relationships with DDx generator companies. Additionally, the use of these tools in the emergency department (ED) setting has not been well studied [3,4].

Importance

DDx tools generate a list of potential diagnoses based on patient history, symptoms, physical findings, and diagnostic data entered by the clinician which may enhance human-generated differentials. Typically, the DDx generated by a diagnosis tool will be much more extensive than that of the clinician, as the tool is not constrained by education, experiential, physiologic, environmental, and recall limitations experienced by human clinicians. Due to the high acuity of patients and lack of follow-up experienced by ED providers, quick and accurate diagnosis is particularly important in this setting to avoid errors that can increase mortality and length of hospital stays [5-7]. DDx tools might have the potential to assist in broadening the DDx and increasing diagnostic precision in the high-acuity environment of the ED [8].

Goals of this investigation

This pilot study aimed to evaluate the utility of a DDx tool newly introduced to an ED setting through analysis of DDx tool clinical usage and a survey of the ED clinicians' perceptions of the tool. The primary outcome of the study was to characterize the usage of the tool by emergency medicine (EM) clinicians based on the findings of the data retrieved from the DDx tool database. The secondary outcome was to assess the clinicians' perceptions of the DDx tool based on the results of a survey.

## Materials and methods

We conducted a mixed-methods analysis, which included both prospective observational data collection and a survey of one ED's experience using a newly introduced DDx-generating tool. The study was conducted at Beaumont Hospital, a high-volume, academic medical center in suburban Detroit, MI, USA with a three-year EM residency training program. The ED serviced an annual volume of approximately 110,000 patients during the study period. Exemption status was granted by the hospital's Institutional Review Board.

The ED provided access to the DDx tool (Isabel Pro, Ann Arbor, MI) to all clinicians working in the department, including attending physicians, resident physicians, and advanced practice clinicians (APCs) in May 2020. The authors had no relationship with the tool company and the sole role of the tool company in the study was to provide de-identified usage data. There was a three-month introductory period during which the clinicians were familiarized with the tool. The study period included the following six months of tool usage in the department. A link to the tool was installed on all desktop computers in the ED and the clinicians were also given access to a mobile format of the tool for use on their personal mobile devices.

The tool uses patient data elements entered by the clinician such as demographics, signs and symptoms, travel history, laboratory data, and imaging data to generate a list of most likely diagnoses. There is no limit on the number of patient data elements that can be entered for a diagnosis query, and more information entered can result in a more specific DDx. Before the initial rollout of the tool, EM clinicians were educated on how to access and use the tool with detailed usage instructions sent out via email as well as a walk-through of the application by one of the study authors at a monthly educational conference with faculty members and residents present. Clinicians were encouraged to use the DDx tool on diagnostically challenging cases. The clinicians were encouraged to re-query the DDx tool as the patient course progressed and more information was collected. The EM clinicians were subsequently sent monthly email reminders of the availability of the tool to encourage continued use.

At the end of the six-month study period, de-identified usage data was provided to the researchers from the DDx tool database for analysis. The search data included the following information: patient age, patient sex, patient pregnancy status, travel history, search query text, and diagnosis output results.

In addition to the usage data collected from the DDx tool database, EM clinicians were requested to complete an anonymous survey sent by email and conducted through REDCap (Research Electronic Data Capture), a secure web-based application designed to support data capture for research studies, to further assess their usage and perception of the tool [9]. The survey was developed by the study authors and edited by a statistician for clarity. This survey included items regarding the clinicians' frequency of use of the DDx tool, characteristics of use, obstacles to use, and perceived utility of the tool in improving various diagnostic and patient safety metrics. Data from both the DDx tool inquiry and survey were analyzed using simple descriptive statistics.

## Results

**Figure 1:**
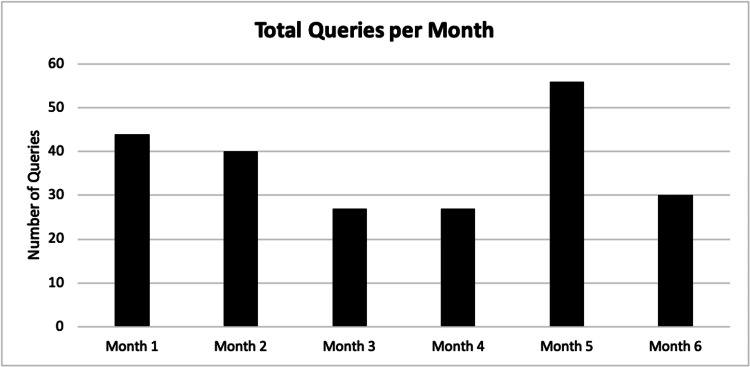
Total queries per month of the study period Usage of the DDx tool declined in the third month of the study period and then did not decline further in subsequent months. Peak usage occurred in the fifth month of the study period. DDx: differential diagnosis

Usage data of the DDx tool included 224 total queries over the six-month study period. Usage dropped in the first three months and then did not drop further in the subsequent three months. Most searches were performed in Month 5 of the study period (Figure 1). A review of the queries revealed that clinicians often entered multiple searches concerning the same patient with varying iterations of the data entered; only the query with the greatest number of data elements was included in the analysis. The tool was used for 0.197% of patients seen in the ED over the study period. There were 224 total queries, which were regarding 107 unique patients as identified by the session ID provided by the DDx tool company. A total of 78.5% of these searches were regarding adult patients and 21.5% were regarding pediatric patients. Forty-four percent of queries were regarding male patients, 54% were regarding female patients, and 1% did not specify sex. Three of the patients were specified as pregnant.

The mean number of patient data elements entered per search was 4.1 with a standard deviation of 3.07. The range of data elements entered was between 1 and 20. The average number of queries carried out per patient was 2.1 with a maximum of 12. A total of 34.6% of searches used lab or radiologic data in the query.

**Figure 2:**
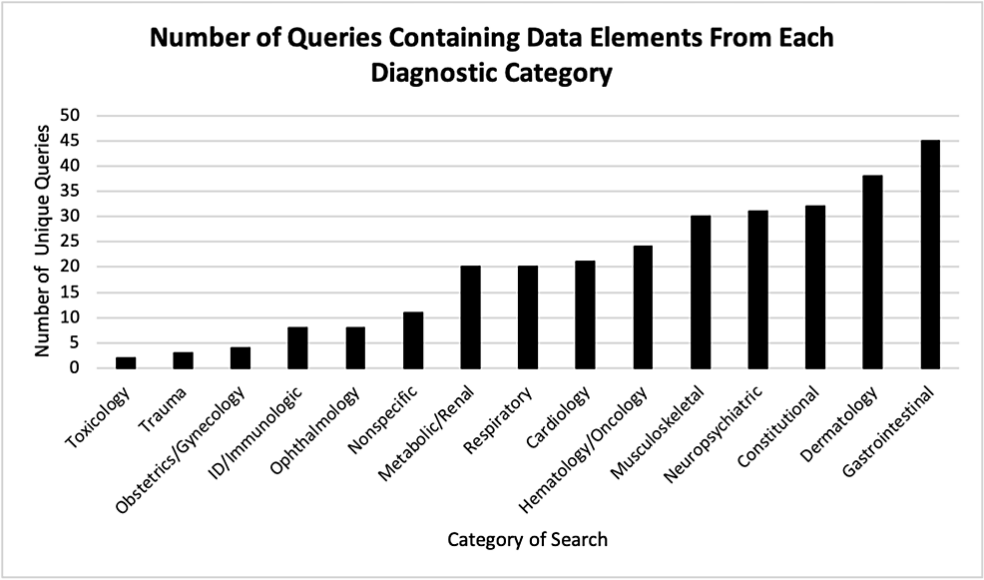
Number of queries containing data elements from each diagnostic category The most commonly queried data elements involved gastrointestinal, dermatologic, and constitutional complaints. Toxicology and trauma complaints were less common.

The results of the data provided by the DDx tool database revealed that the cases most commonly searched by clinicians involved dermatologic, gastrointestinal, and constitutional data elements. The least commonly searched data elements were related to toxicology and trauma (Figure 2).

The survey was sent to 120 ED clinicians, including 28 APCs, 42 attending physicians, and 50 resident physicians. Of the 32 clinicians who responded to the survey, only seven reported using the DDx tool. The most commonly reported reasons for not using the tool were forgetting that the tool was available for use, perception of low utility, and disruption to workflow.

## Discussion

The ED is a hospital setting where clinicians must rapidly evaluate, diagnose, and treat potentially critically ill patients, often with confusing or little information on the patient's history. An electronic DDx tool may have the potential to be of benefit in supporting accurate diagnosis in the ED by providing an extensive DDx which would be otherwise difficult for the typical ED clinician to generate from memory and experience. However, most current published research on the use of DDx tools is from non-clinical and primary care office-based settings and it is unclear if the benefits seen in those studies translate to the ED given the unique challenges of the setting [1,2]. Currently, there is limited data on the value of the electronic DDx tool in the ED environment.

ED clinicians in our study entered an average of 4.1 data elements per patient into the DDx tool. Clinicians often also re-queried the tool on the same patient as they obtained more data. Notably, clinicians input data on laboratory or radiologic findings in only one-third of patients, which potentially limited the DDx tool's output. Given that the DDx tool places no limit on the number of data elements that can be input, education of EM clinicians on the tool's capacity to assess large numbers of data elements, including laboratory and radiologic data, may increase the tool's utility in the ED.

EM clinicians in our study utilized the DDx tool most frequently for patient presentations involving constitutional, dermatologic, and gastroenterological symptoms. This likely reflects diagnostic uncertainty experienced by the clinicians due to the large variety of potential diagnoses that a patient presenting with these symptom complexes could have. However, it is possible that existing tools that are specific to dermatologic cases and which allow visual input may be more useful for dermatologic presentations.

For presentations relating to toxicology and trauma, the DDx tool was rarely used. In the case of toxicologic presentations, it is frequently known what the exposure was, eliminating diagnostic uncertainty. Furthermore, EM clinicians in the United States have the resources of Poison Control Centers to assist in their evaluation of complex toxicologic cases and may not utilize a DDx tool in this setting. Trauma patients are managed by the protocols taught in Advanced Trauma Life Support courses, which direct the diagnostic evaluation through standardized physical exams and diagnostic studies, and therefore make the DDx tool less useful in the trauma setting.

Despite encouragement and multiple reminders from the study team to the ED staff to maximize EM provider utilization of the DDx tool, the tool was used by a relatively low number of clinicians during the study period. The reasons cited for not using the tool included forgetting that the tool was available for use, disruption to workflow, and perception of low utility. The hesitance to use the tool among clinicians due to a perceived lack of need is consistent with studies that have shown that the diagnostic confidence of clinicians is independent of both the difficulty of the case and the accuracy of their diagnosis [10]. Additionally, EM clinicians are often unaware of diagnostic errors they may have committed due to a lack of patient continuity in the ED setting, potentially resulting in false confidence in their diagnostic skill set and a reluctance to use a diagnostic aid [5]. Future research on the ED use of a DDx tool is needed to better understand ways to more seamlessly integrate DDx tools into the EM clinician’s workflow. Additionally, further investigation may be helpful in evaluating if such tools can improve diagnostic accuracy and patient outcomes in the ED environment.

Our study had several limitations. First, it was conducted at a single tertiary-care, academic ED, and the results may not be applicable to smaller centers or to non-academic environments. Also, in our ED, clinicians utilized the DDx tool on a voluntary basis. Had tool usage been required or automated, a better understanding of the DDx tool’s abilities could be more fully understood. Due to the low response to the survey, it is unclear if the tool has more utility for APCs, EM residents, or seasoned EM physicians. Additionally, usage data from the DDx tool database was de-identified which limited the ability to analyze usage by individuals who did not respond to the survey or ascertain the exact number of unique clinicians who used the tool. Finally, as already noted, self-reported usage of the tool by survey respondents was exceptionally low, limiting the ability to subjectively characterize tool usage.

## Conclusions

In summary, this pilot study found that DDx tools may have some utility in the ED setting, particularly for cases involving dermatologic, gastrointestinal, and constitutional complaints. However, clinician adoption and integration of DDx tools into clinicians' workflow are barriers to their use. Further research is needed to investigate methods to reduce these limitations before the utility of DDx tools in this setting can be fully elucidated.

## References

[1] Riches N, Panagioti M, Alam R, Cheraghi-Sohi S, Campbell S, Esmail A, Bower P (2016). The effectiveness of electronic differential diagnoses (DDX) generators: a systematic review and meta-analysis. PLoS One.

[2] Henderson EJ, Rubin GP (2013). The utility of an online diagnostic decision support system (Isabel) in general practice: a process evaluation. JRSM Short Rep.

[3] Berry AC, Cash BD, Wang B (2019). Online symptom checker diagnostic and triage accuracy for HIV and hepatitis C. Epidemiol Infect.

[4] Ramnarayan P, Roberts GC, Coren M (2006). Assessment of the potential impact of a reminder system on the reduction of diagnostic errors: a quasi-experimental study. BMC Med Inform Decis Mak.

[5] Schiff GD (2008). Minimizing diagnostic error: the importance of follow-up and feedback. Am J Med.

[6] Croskerry P, Sinclair D (2001). Emergency medicine: a practice prone to error?. CJEM.

[7] Hautz WE, Kämmer JE, Hautz SC (2019). Diagnostic error increases mortality and length of hospital stay in patients presenting through the emergency room. Scand J Trauma Resusc Emerg Med.

[8] Abimanyi-Ochom J, Bohingamu Mudiyanselage S, Catchpool M, Firipis M, Wanni Arachchige Dona S, Watts JJ (2019). Strategies to reduce diagnostic errors: a systematic review. BMC Med Inform Decis Mak.

[9] Harris PA, Taylor R, Thielke R, Payne J, Gonzalez N, Conde JG (2009). Research electronic data capture (REDCap) - a metadata-driven methodology and workflow process for providing translational research informatics support. J Biomed Inform.

[10] Meyer AND, Payne VL, Meeks DW, Rao R, Singh H (2013). Physicians' diagnostic accuracy, confidence, and resource requests: a vignette study. JAMA Intern Med.

